# Supplementary material for: Hepatocyte-specific deletion of Pparα promotes NAFLD in the context of obesity
Source: Sci Rep. 2020 Apr 16;10:6489. doi: 10.1038/s41598-020-63579-3 (PMC7162950; doi:10.1038/s41598-020-63579-3)
Supplement: Supplementary file 1 — Supplementary information. [file 41598_2020_63579_MOESM1_ESM.pdf]

Supplementary Informations

Hepatocyte-specific deletion of *Pparα* promotes NAFLD in the context of obesity

Marion Régnier[1], Arnaud Polizzi[1], Sarra Smati[1,2], Céline Lukowicz[1], Anne Fougerat[1], Yannick Lippi[1], Edwin Fouché[1], Frédéric Lasserre[1], Claire Naylies[1], Colette Bétoulières[1], Valentin Barquissau[2], Etienne Mouisel[2], Justine Bertrand-Michel[3], Aurélie Batut[3], Talal Al Saati[4], Cécile Canlet[1], Marie Tremblay-Franco[1], Sandrine Ellero-Simatos[1], Dominique Langin[2,5], Catherine Postic[6], Walter Wahli[1,7,8] Nicolas Loiseau[1], Hervé Guillou[1*], Alexandra Montagner[1,2*]

1. Toxalim, INRAE UMR 1331, ENVT, INP-Purpan, University of Toulouse, Paul Sabatier University, F-31027 Toulouse, France

2. Institut National de la Santé et de la Recherche Médicale (INSERM), UMR1048, Institute of Metabolic and Cardiovascular Diseases, University of Toulouse, Paul Sabatier University, Toulouse, France

3. Metatoul-Lipidomic Facility, MetaboHUB, Institut National de la Santé et de la Recherche Médicale (INSERM), UMR1048, Institute of Metabolic and Cardiovascular Diseases, Toulouse, France

4. Service d'Histopathologie Expérimentale Unité INSERM/UPS/ENVT-US006/CREFRE Inserm, CHU Purpan, 31024 Toulouse cedex 3, France

5. Toulouse University Hospitals, Laboratory of Clinical Biochemistry, Toulouse, France

6. Institut National de la Santé et de la Recherche Médicale (INSERM), U1016, Institut Cochin, Paris, France

7. Lee Kong Chian School of Medicine, Nanyang Technological University Singapore, Clinical Sciences Building, 11 Mandalay Road, Singapore

8. Center for Integrative Genomics, Université de Lausanne, Le Génopode, Lausanne, Switzerland

Supplementary Table 1. Oligonucleotide sequences for real-time qPCR. Oligonucleotides (Sigma-Aldrich) were designed using Primer Express 2.0 software (Applied Biosystems). Couples of primers have a Tm of 60°C, exhibit no amplification with genomic DNA, and the derivative from each dissociation curve of amplicons from cDNA exhibits a single specific peak.

Gene	NCBI Refseq	Forward primer (5'-3')	Reverse primer (5'-3')
Col1a1	NM_007742	GGCTCCTGCTCCTCTTAGGG	TCGGGTTTCCACGTCTCAC
Fmo3	NM_008030	AAGAAAGGAAGACAAAGAAAAGGCA	AGCTCCAATGATGGCCACTT
Ppar-γ2	NM_011146	GATGCACTGCCTATGAGCACTT	GAATGGCATCTCTGTGTCAACC
Vnn1	NM_011704	ATGAGGTTTATGCCTTTGGAGC	CCACAGGTGCGTAAATTGGTAG

a

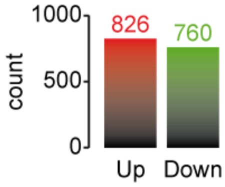

b

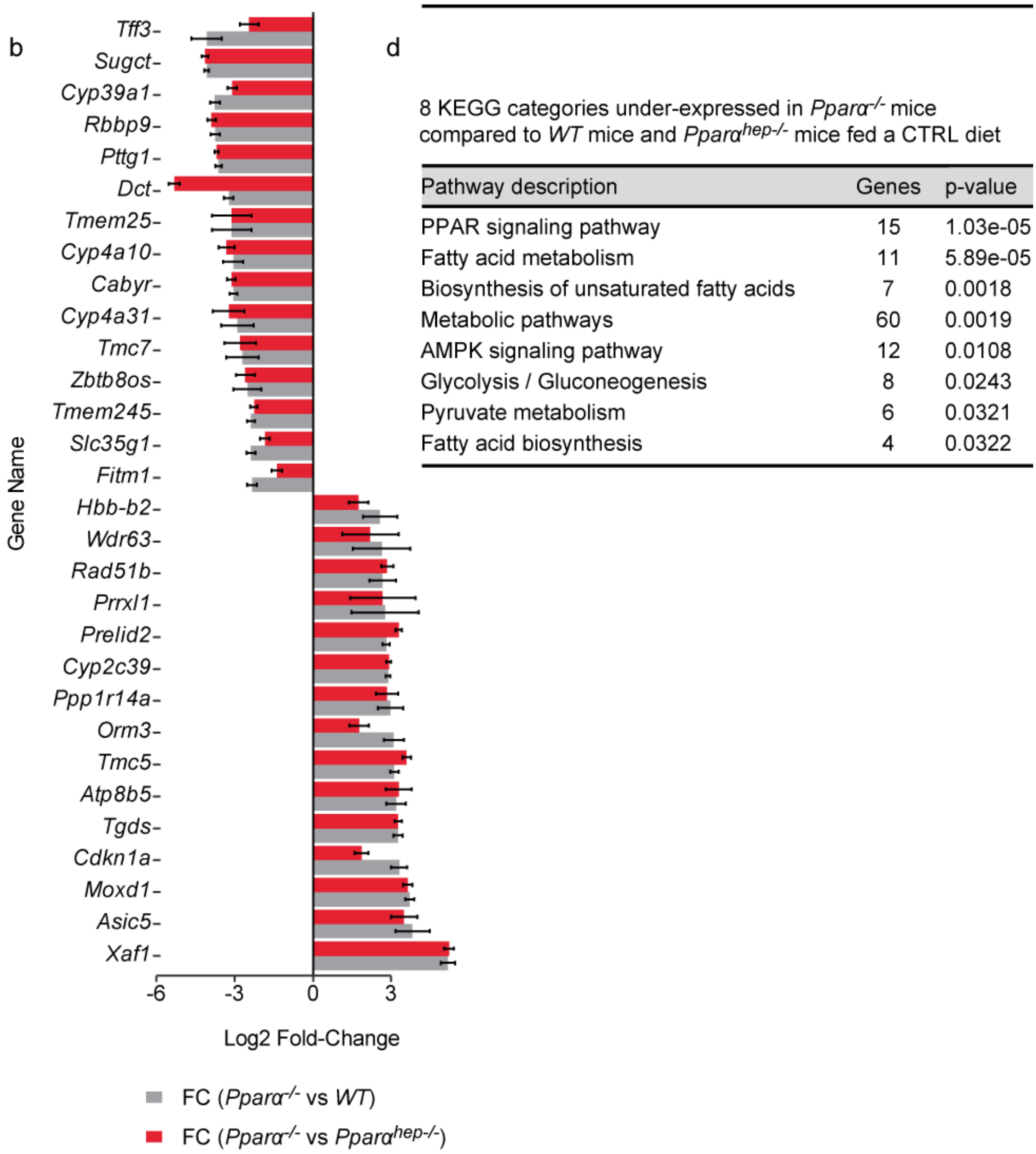

c

2 KEGG categories over-expressed in *Pparα*$^{-/-}$mice compared to *WT* mice and *Pparα*$^{hep-/-}$ mice fed a CTRL diet

Pathway description	Genes	p-value
Glioma	10	0.0098
Melanoma	9	0.0328

d

8 KEGG categories under-expressed in *Pparα*$^{-/-}$ mice compared to *WT* mice and *Pparα*$^{hep-/-}$ mice fed a CTRL diet

Pathway description	Genes	p-value
PPAR signaling pathway	15	1.03e-05
Fatty acid metabolism	11	5.89e-05
Biosynthesis of unsaturated fatty acids	7	0.0018
Metabolic pathways	60	0.0019
AMPK signaling pathway	12	0.0108
Glycolysis / Gluconeogenesis	8	0.0243
Pyruvate metabolism	6	0.0321
Fatty acid biosynthesis	4	0.0322

Supplementary Figure 1. Differential hepatic gene expression profiles by comparing *Pparα*$^{-/-}$ to both *WT* and *Pparα*$^{hep-/-}$ mice. (a) Number of differentially expressed genes by comparing *Pparα*$^{-/-}$ to both WT and *Pparα*$^{hep-/-}$ mice (adjusted p≤0.05) (b) Grey bars represent the top 15 induced and repressed genes specifically in *WT* mice compared to *Pparα*$^{-/-}$ mice fed a CTRL diet. Red bars represent the differentially expressed genes between *Pparα*$^{hep-/-}$ and *Pparα*$^{-/-}$ mice fed a CTRL diet (p≤0.05). (c) Analysis of KEGG categories over-expressed in Pparα-/-mice compared to both WT mice and *Pparα*$^{hep-/-}$ mice fed a CTRL diet (p≤0.05). (d) Analysis of KEGG categories under-expressed in *Pparα*-/-mice compared to both WT mice and *Pparα*$^{hep-/-}$ mice fed a CTRL diet (p≤0.05).

a

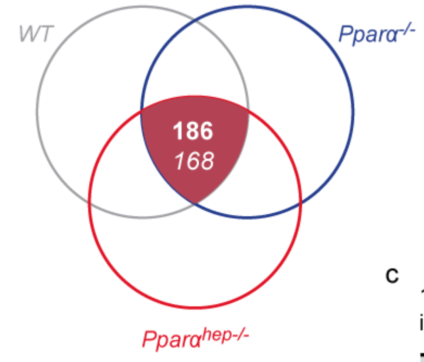

b

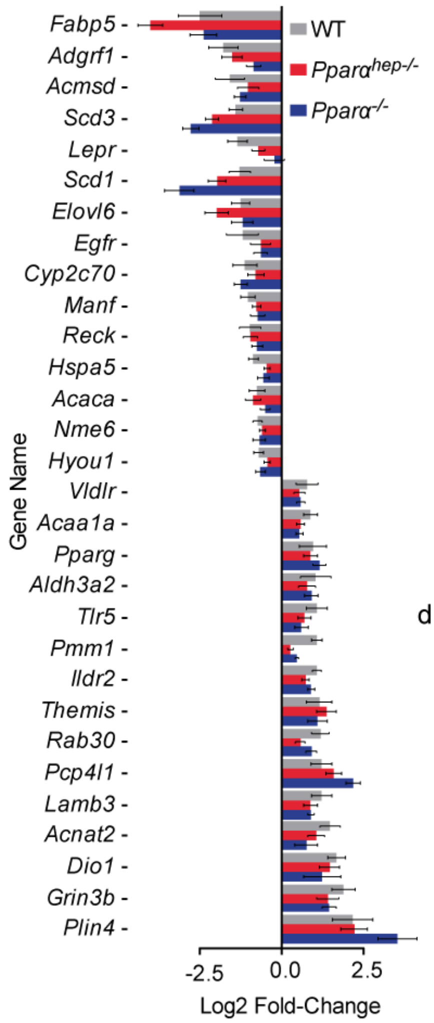

c

15 KEGG categories up-regulated in response to HFD in *WT*, *Pparα*$^{-/-}$ and in *Pparα*$^{hep-/-}$

Pathway description	Genes	p-value
Metabolic pathways	37	7.35e-12
Fatty acid degradation	10	6.4e-11
Butanoate metabolism	7	3.48e-08
Fatty acid metabolism	8	1.35e-07
Propanoate metabolism	6	3.65e-06
Microbial metabolism	10	1.61e-05
Ketone bodies metabolism	4	1.81e-05
Carbon metabolism	7	0.000378
PPAR signaling pathway	6	0.000579
Peroxisome	6	0.000654
Beta-Alalanine metabolism	4	0.00161
Pyruvate metabolism	4	0.00497
Tryptophan metabolism	4	0.00554
Renin-angiotensin system	3	0.00604
Lysine degradation	4	0.00858

d

3 KEGG categories down-regulated in response to HFD in *WT*, *Pparα*$^{-/-}$ and in *Pparα*$^{hep-/-}$

Pathway description	Genes	p-value
Protein processing in ER	17	3.27e-13
Protein export	8	3.24e-10
Fatty acid metabolism	6	0.002

Supplementary Figure 2. Common changes in the hepatic gene expression profiles of *WT*, *Pparα*$^{hep-/-}$, *Pparα*$^{-/-}$ mice in response to HFD. (a) Venn diagram presenting the number of overlapping hepatic genes up-regulated (bold) and down-regulated (regular) in response to HFD in *WT*, *Pparα*$^{hep-/-}$, and *Pparα*$^{-/-}$ mice (adjusted p≤0.05) (b) Grey bars represent the top 15 induced and repressed genes specifically in *WT* mice exposed to CTRL diet vs HFD. Red and blue bars represent the HFD-induced responses in *Pparα*$^{hep-/-}$ and *Pparα*$^{-/-}$, respectively. (c) Analysis of KEGG categories up-regulated in *WT*, *Pparα*$^{hep-/-}$ and *Pparα*$^{-/-}$ mice fed a HFD vs. CTRL diet (p≤0.01). (d) Analysis of KEGG categories down-regulated in *WT*, *Pparα*$^{hep-/-}$ and *Pparα*$^{-/-}$ mice fed a HFD (p≤0.01).

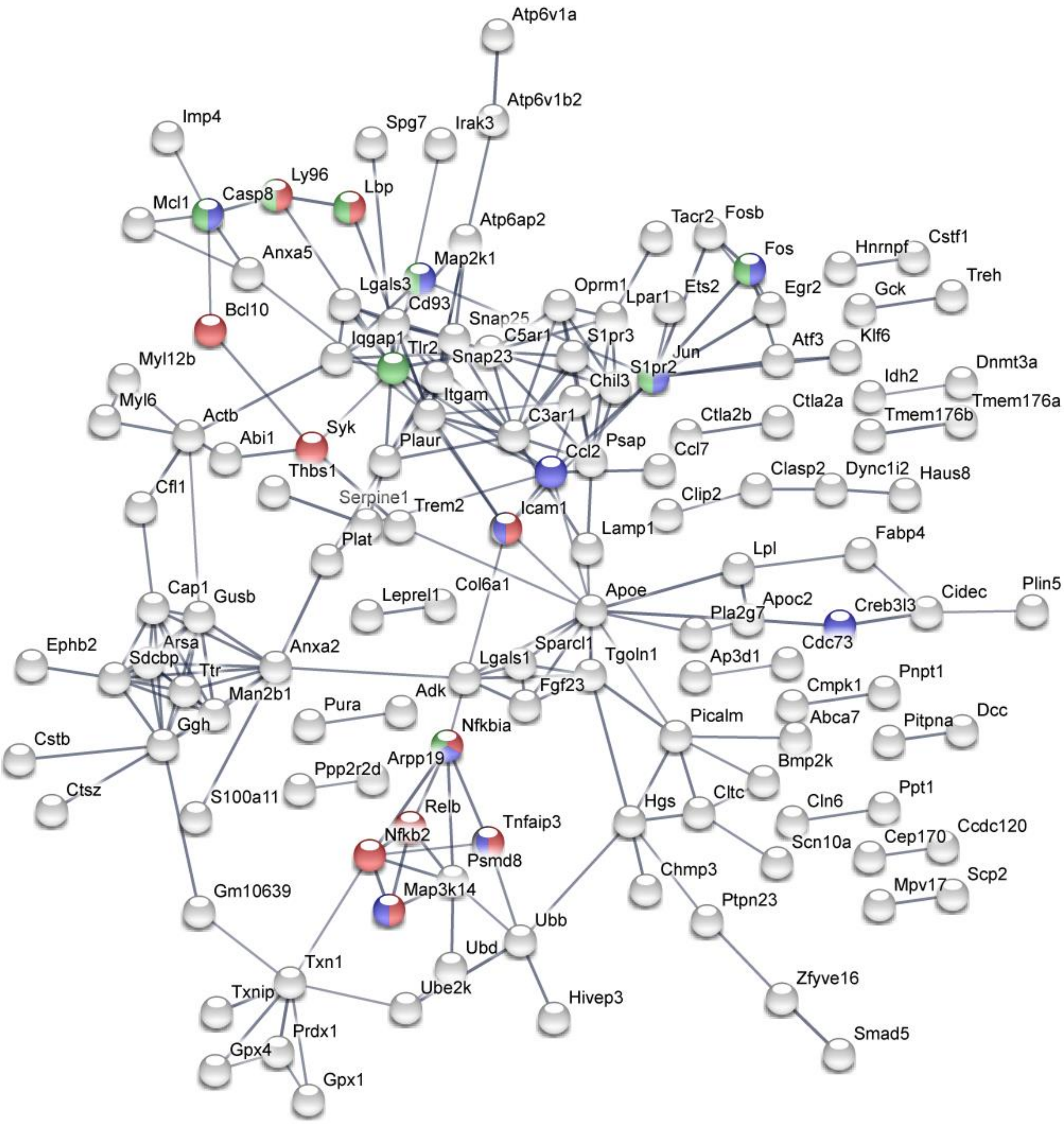

Supplementary Figure 3. Identification of genes related to NF-kappa B, TNF, and TLR signalling pathways. The predicted gene-gene interaction network (Search Tool for the Retrieval of Interacting Genes/StringV10) among genes significantly induced in $Ppar\alpha^{hep-/-}$, $Ppar\alpha^{-/-}$ mice (adjusted p≤0.05) fed a HFD. Genes coloured in red, blue, and green belong to the GO categories "NF-kappa B signalling pathway", "TNF signaling pathway", and "TLR signalling pathway" respectively.

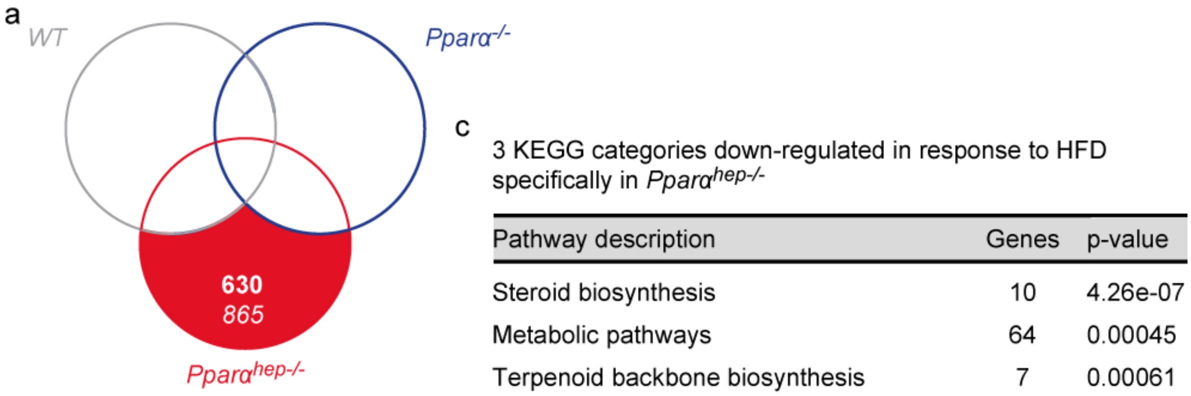

c

3 KEGG categories down-regulated in response to HFD specifically in *Pparα*$^{hep-/-}$

Pathway description	Genes	p-value
Steroid biosynthesis	10	4.26e-07
Metabolic pathways	64	0.00045
Terpenoid backbone biosynthesis	7	0.00061

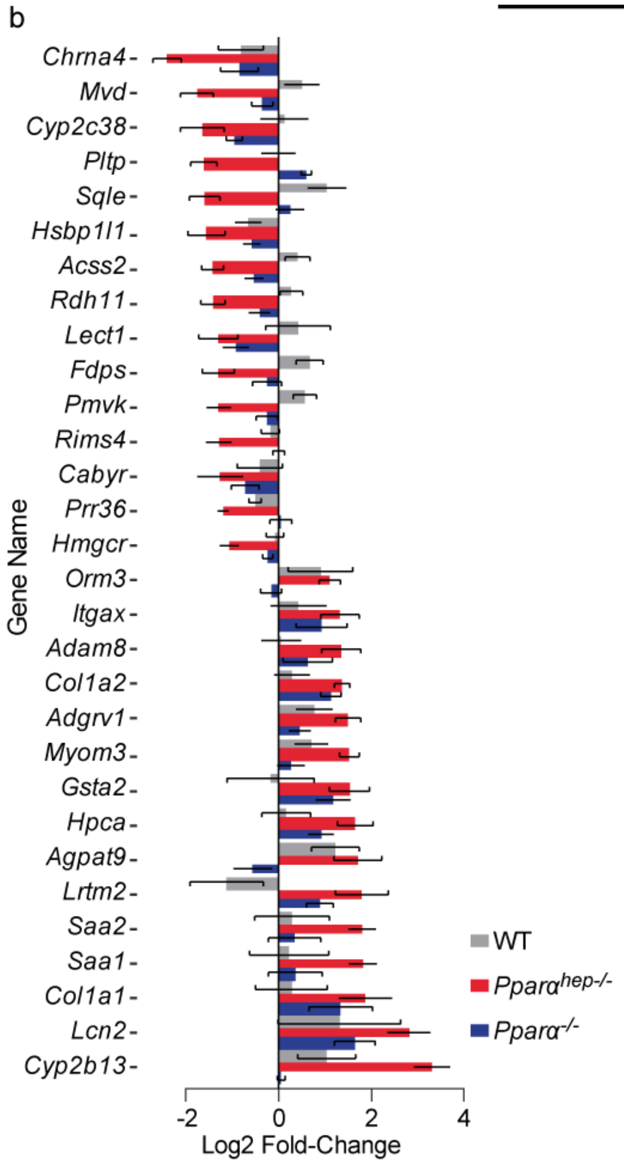

Supplementary Figure 4. Specific changes in liver gene expression profiles in *Pparα*$^{hep-/-}$ mice fed a HFD. (a) Venn diagram presenting the number of hepatic genes up-regulated (bold) and down-regulated (regular) in response to HFD in *WT*, *Pparα*$^{hep-/-}$ and *Pparα*$^{-/-}$ mice (adjusted p-value; p≤0.05) (b) Red bars represent the top 15 induced and repressed genes in *Pparα*$^{hep-/-}$ mice fed a HFD vs CTRL diet. Grey bars represent the gene expression profile in WT mice and blue bars the gene expression profile in *Pparα*$^{-/-}$ mice (c) Analysis of KEGG categories specifically down-regulated in *Pparα*$^{hep-/-}$ mice fed a HFD (p≤0.01).

a

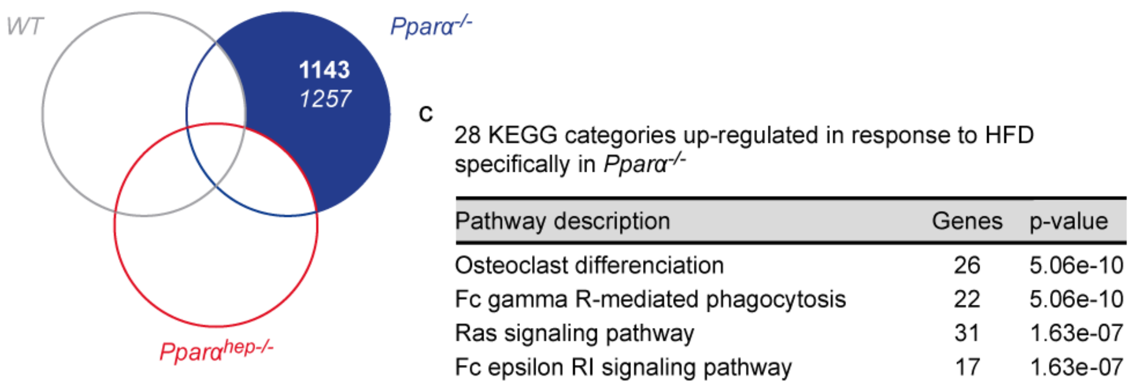

b

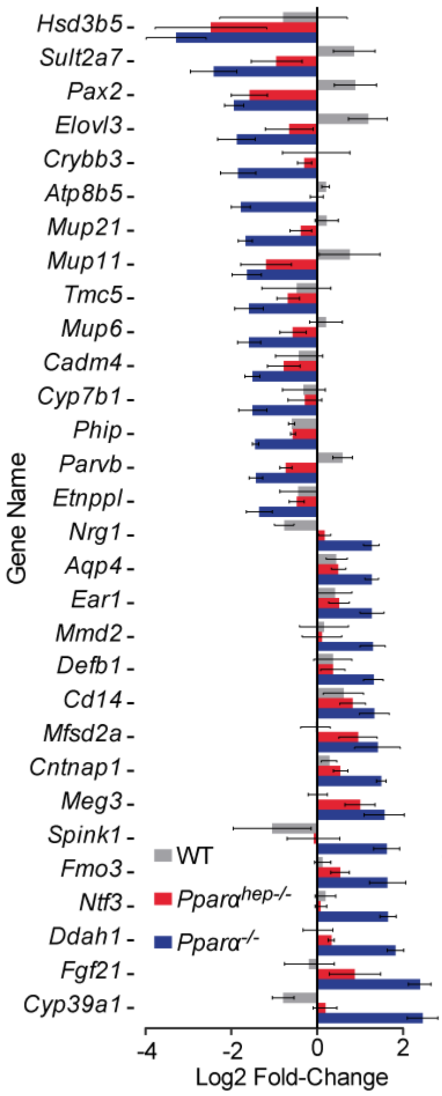

c

28 KEGG categories up-regulated in response to HFD specifically in Pparα-/-

Pathway description	Genes	p-value
Osteoclast differenciation	26	5.06e-10
Fc gamma R-mediated phagocytosis	22	5.06e-10
Ras signaling pathway	31	1.63e-07
Fc epsilon RI signaling pathway	17	1.63e-07
MAPK signaling pathway	32	7.05e-07
Chemokine signaling pathway	25	3.32e-06
Toll-like receptor signaling pathway	18	4.79e-06
Natural killer cell mediated cytotoxicity	19	5.8e-06
Staphylococcus aureus infection	12	1.92e-05
Leishmaniasis	13	4.59e-05
Eipstein-Barr virus infection	24	5.58e-05
Rap1 signaling pathway	24	0.000106
PI3K-Akt signaling pathway	33	0.000106
Pertussis	13	0.000106
Viral myocarditis	13	0.000106
Hepatitis B	19	0.000114
HIF-1 signaling pathway	16	0.000124
Leukocyte transendothelial migration	17	0.000144
Viral carcinogenesis	22	0.000314
Tuberculosis	20	0.000329
ErbB signaling pathway	13	0.000488
HTLV-I infection	26	0.000497
Cell adhesion molecules (CAMs)	18	0.000633
Pancreatic cancer	11	0.000633
Malaria	9	0.000699
Chagas disease	14	0.000785
Platelet activation	16	0.000827
Amoebiasis	15	0.000998

d

1 KEGG categories down-regulated in response to HFD specifically in Pparα-/-

Pathway description	Genes	p-value
RNA degradation	12	0.00941

Supplementary Figure 5. Specific changes in liver gene expression profiles in *Pparα*-/- mice fed a HFD. (a) Venn diagram presenting the number of hepatic genes up-regulated (bold) and down-regulated (regular) in response to HFD in *WT*, *Pparα*hep-/- and *Pparα*-/- mice (adjusted p≤0.05). (b) Blue bars represent the top 15 induced and repressed genes in *Pparα*-/- mice fed a HFD vs. CTRL diet. Grey bars represent the gene expression profile in *WT* mice and red the gene expression profile in *Pparα*hep-/- mice (c) Analysis of KEGG categories specifically up-regulated in *Pparα*-/- mice fed a HFD (p≤0.001).

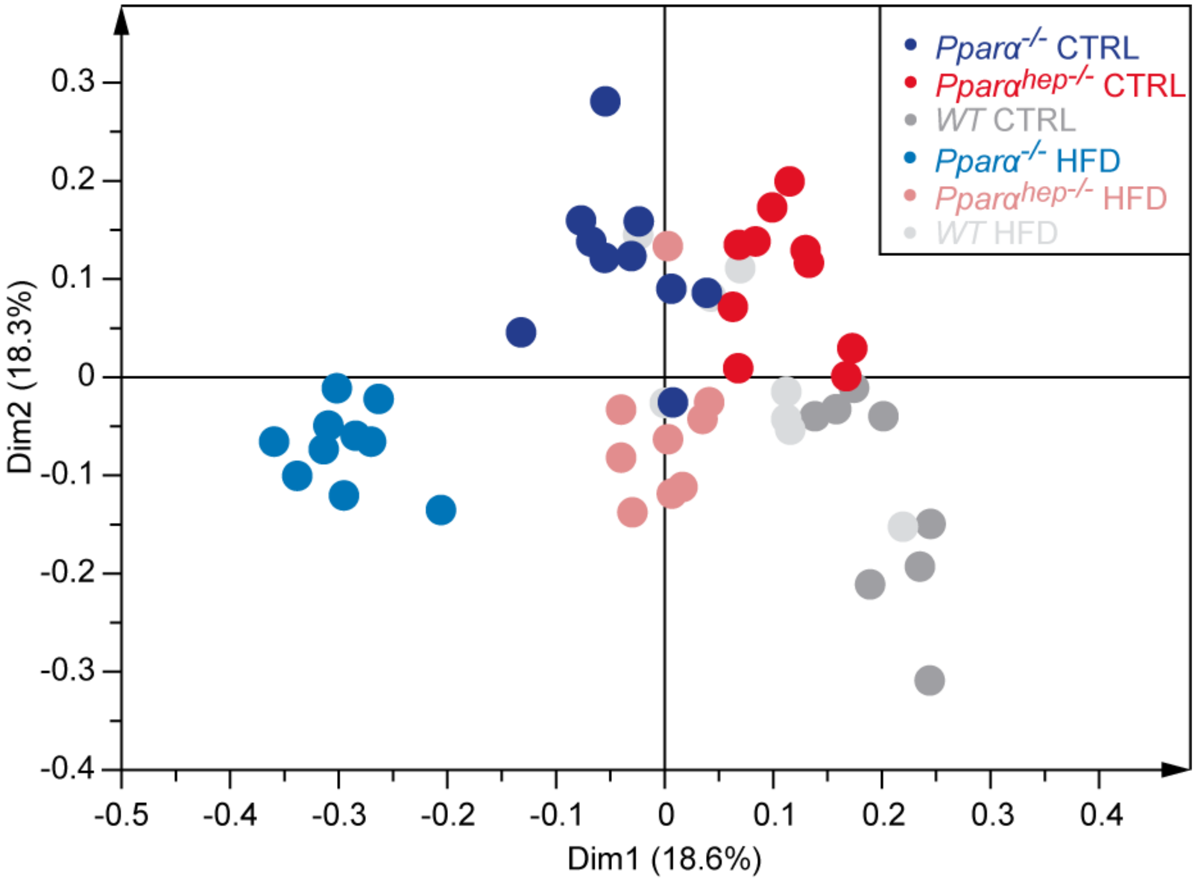

Supplementary Figure 6. Liver metabolome discriminates whole-body *Pparα* deletion from hepatocyte-specific PPARα deletion in mice fed a HFD. Two-dimensional PLS-DA score plot of liver extract integrated 1H-NMR spectra. Each dot represents an observation (animal), projected onto first (horizontal axis) and second (vertical axis) PLS-DA variables.
